# Editorial: The State-of-Art in Immuno-Oncology, What to Do With Glioblastoma?

**DOI:** 10.3389/fimmu.2021.788733

**Published:** 2021-10-27

**Authors:** Xiaoteng Cui, Qixue Wang, Chunsheng Kang

**Affiliations:** Lab of Neuro-oncology, Tianjin Neurological Institute, Key Laboratory of Post-Neuroinjury Neuro-repair and Regeneration in Central Nervous System, Department of Neurosurgery, Tianjin Medical University General Hospital, Tianjin, China

**Keywords:** glioblastoma, immunotherapy, tumor microenvironment, CAR T cell therapy, multi-target

Gliomas are the most common primary tumors of the central nervous system, originating in the brain which is one of the immunologically privileged organs. As high-grade glioma accounts for 80% of malignant brain tumors, glioblastomas (GBM) are characterized by high inter- and intra-tumoral heterogeneity and immunosuppressive tumor microenvironment. At present, the conventional treatment of GBM in a clinical context is surgical resection, combined with systematic radio- and chemo-therapy. But the median survival time of GBM patients is still not ideal. Therefore, an urgent need for finding new and more effective treatment strategies remains.

In recent years, some novel therapeutic strategies, such as immunotherapies represented by checkpoint inhibitors/antibodies and cell therapies exemplified by chimeric antigen receptor (CAR) engineered T cells, give new hope for current GBM patients. These novel therapeutics targeted T cells aiming to improve their infiltration and enhance the activity of cytotoxic T cells. However, many factors in the tumor microenvironment can prevent T cell infiltration and induce T cells to the exhausted subtype, such as tumor-associated macrophages that form immune barriers and metabolites secreted by tumor cells. Hence, elucidating the characteristics of the GBM immune microenvironment is crucial for immunotherapies for GBM.

The Research Topic “The State-of-Art in Immuno-oncology, what to do with Glioblastoma?” focuses on every aspect of immunology in GBM, aiming to clarify the immune landscape of GBM and bridge the immunology and therapeutics of GBM.

Yuanhao Chang et al. reported that a gene named Glutaredoxin (GLRX), which is a vital gene maintaining cell redox balance and playing a regulatory role in the progression of many malignant tumors, could be highly expressed in high-grade gliomas, and become an independent prognostic predictor of GBM patients. Mechanically, by employing the single-cell RNA sequencing of GBM samples, and bioinformatic analysis of CGGA and TCGA online databases, the authors demonstrated the GLRX was specifically expressed in M0 macrophages, and positively correlated with the complexity of tumor microenvironment and the infiltrated immune cells, indicating that GLRX was a key regulator of glioma immune microenvironment formation. Therefore, therapeutics targeting the cell redox regulation are expected to enhance the effect of glioma immunotherapy.

It is well-known that GBM is a malignant tumor with a high recurrence rate. Resistance to various therapeutics often occurs in recurrent GBM, with the remodeling of the immunosuppressive microenvironment. Weilun Fu et al. presented a high-dimensional view of the complex immune microenvironment in primary and recurrent GBM by mass cytometry (CyTOF). They demonstrated that glioma-associated macrophages (GAMs) accounted for a large proportion of GBM tumors, exhibiting great inter- and intra-tumoral heterogeneity. GAMs and other immune cells such as exhausted T cells, infiltrating Tregs, and nonfunctional NK cells contributed to the immunosuppressive characteristics. Primary and recurrent GBM showed a similar immune microenvironment, but the proportion of GAMs decreased from 59.05% in primary GBM to 27.87% in recurrent GBM. These results above suggest to us that more specific and comprehensive therapeutic strategies are urgently needed to treat the recurrent GBM.

Recently, CAR T cell therapy directed at tumor-specific targets has achieved great effects in the treatment of a variety of tumors. But the response to CAR T therapy in GBM is debatable. Long Li et al. reviewed the current status and prospects of CAR T immunotherapy in GBM treatment and found that CAR T cells targeting IL-13RA2, EGFRvIII, and HER2 showed significant clinical efficacy and safety in phase 1 and 2 clinical trials conducted in patients with GBM. However, its efficacy is still limited by the blood-brain barrier, high tumoral heterogeneity, and antigen escape. Therefore, the combined therapeutic strategy of conventional therapy and multi-target CAR T cells is more appropriate for GBM treatment. A review on glioma immunotherapy by Boyuan Huang et al. presented the same viewpoints. Meanwhile, they pointed out that combined therapeutics such as vaccine research and development, immune checkpoint blocking, and CAR T cell targeting have the prospect of glioma immunotherapy.

Recent evidence has indicated exosomes and metabolites from glioma cells could remold the immune-microenvironments within the tumor community. The lipid and cholesterol metabolic by-pathway could reinforce such an effect. The future battlefield will be a multi-target approach both at glioma cells and immune cells. Alternative therapeutic avenues including innovative targets and drug and effective antibody delivery methods will shed new light on GBM therapy.

## Author Contributions

All authors have contributed to the manuscript and approved the final version for publishment.

## Conflict of Interest

The authors declare that the research was conducted in the absence of any commercial or financial relationships that could be construed as a potential conflict of interest.

## Publisher’s Note

All claims expressed in this article are solely those of the authors and do not necessarily represent those of their affiliated organizations, or those of the publisher, the editors and the reviewers. Any product that may be evaluated in this article, or claim that may be made by its manufacturer, is not guaranteed or endorsed by the publisher.

